# Curcumin enhances the effectiveness of cisplatin by suppressing CD133^+^ cancer stem cells in laryngeal carcinoma treatment

**DOI:** 10.3892/etm.2013.1297

**Published:** 2013-09-13

**Authors:** HEJIA ZHANG, TIANYU YU, LIANJI WEN, HUI WANG, DAN FEI, CHUNSHUN JIN

**Affiliations:** 1 Department of Otorhinolaryngology, Second Hospital of Jilin Uinversity, Changchun, Jilin 130041;; 2 Departments of Ultrasonography, China-Japan Union Hospital of Jilin University, Changchun, Jilin 130033, P.R. China; 3 Thyroid Surgery, China-Japan Union Hospital of Jilin University, Changchun, Jilin 130033, P.R. China

**Keywords:** curcumin, cisplatin, cancer stem cells, laryngeal carcinoma

## Abstract

Chemoresistance is one of the major barriers to chemotherapeutic treatment and it has been established that CD133^+^ cancer stem cells are responsible for drug resistance in laryngeal carcinoma. In the present study, curcumin and cisplatin were used as a combined treatment to induce the sensitivity of CD133^+^ cancer stem cells to chemotherapeutic agents and to enhance therapeutic effectiveness. The results revealed that in untreated and cisplatin-treated HEp-2 cell groups, the percentage of CD133^+^ cells was 4.50 and 6.89%, respectively. However, in the combined treatment group, the percentage of CD133^+^ cells was markedly reduced to 1.49%, indicating that curcumin may increase the sensitivity of CD133^+^ cells to cisplatin, leading to the suppression of chemoresistance in HEp-2 cells. Furthermore, the expression of ATP-binding cassette sub-family G member 2 (ABCG2), which is an important gene for chemoresistance, was demonstrated to be reduced in CD133^+^ cancer stem cells following combined treatment. These results suggest that the combined application of curcumin with chemotherapeutic drugs may be a reliable and effective approach for the treatment of laryngeal carcinoma.

## Introduction

The resistance of tumor cells to chemotherapeutic agents is one of the major barriers suppressing the effectiveness of anticancer drugs in cancer treatment. It has been demonstrated that cancer stem cells, which are important in carcinogenesis, tumor progression, invasion and metastasis, are also responsible for chemoresistance (1). *In vitro* studies revealed that stem-like cells from breast cancer cell lines were less sensitive to paclitaxel and 5-fluorouracil (2), and a side population of cells in hepatocellular carcinoma (HCC) exhibited resistance to doxorubicin (3).

We have previously reported that in the laryngeal carcinoma cell line HEp-2, ~1.5–3.5% of cells were CD133^+^ cancer stem cells, which are responsible for chemoresistance to cisplatin (3,4). CD133^+^ cancer stem cells may be enriched after cisplatin treatment, leading to chemoresistance (5,6). In addition to chemoresistance, platinum-based chemotherapy may have other dose-dependent side-effects that limit the application of platinum-based drugs in cancer treatment (7). As platinum-based chemotherapy is currently widely used to treat a number of cancer types, particularly head and neck cancers (8), it is necessary to develop an optimal therapy to reduce chemoresistance, suppress tumor recurrence and enhance effectiveness.

Curcumin (diferuloylmethane) is extracted from the rhizome of *Curcuma Longa*, which has been used as a traditional Chinese medicine for centuries (9). Curcumin is also the major component of turmeric, a spice widely used in Asian cuisines. Previous studies have revealed that curcumin has protective and anti-cancer effects in several types of human cancer (10). Curcumin may function through a series of signaling pathways implicated in cancer development and, since it is a promising anticancer drug, it is important to evaluate the beneficial effect of curcumin as a single treatment or when administered in combination with other conventional anticancer drugs (11). As curcumin is insoluble in water, any form of inorganic solution or saline, previous studies have focused on liposomal formulations that may aid the delivery of curcumin (12).

In the present study, we investigated the anticancer effect of treatment with a combination of curcumin and cisplatin. The ability of curcumin to enhance the effectiveness of cisplatin in HEp-2 cells and induce the sensitivity of CD133^+^ cancer stem cells to cisplatin by suppressing ATP-binding cassette sub-family G member 2 (ABCG2)-mediated chemoresistance *in vitro* was evaluated.

## Materials and methods

### Chemicals and reagents

Cisplatin, 3-(4,5-dimethylthiazol-2-yl)-2,5-diphenyltetrazolium bromide (MTT), 1,2-dimyristoyl-sn-glycero-3-phosphocholine (DMPC) and 1,2-dimyristoyl-sn-glycero-3-[phospho-rac-(1-glycerol)] (DMPG) were purchased from Sigma-Aldrich (St. Louis, MO, USA). Curcumin was obtained from Dingguo Biotech Inc. (Beijing, China). The glycine buffer used in the MTT assay was prepared with 0.1 M glycine and 0.1 M NaCl, and adjusted to pH 10.5.

### Liposomal curcumin preparation

A lipid mixture was prepared by mixing DMPC and DMPG in a ratio of 9:1. Curcumin was added to the lipid mixture with sterile water to create a liposomal curcumin solution with a final lipid:curcumin ratio of 10:1. The liposomal curcumin solution was then filtered and lyophilized. The lyophilized liposomal curcumin was suspended with 0.9% NaCl to achieve a stock concentration of 100 mmol/l.

### Cell culture and treatment

A human laryngeal squamous cancer cell line, HEp-2, was provided by the Second Hospital of Jilin University (Changchun, China). Cells were cultured in Dulbecco’s modified Eagle’s medium with 10% fetal bovine serum (FBS) at 37°C in a 5% CO_2_ incubator. 100 U/ml penicillin and 100 *μ*g/ml streptomycin were used to prevent microbial contamination. Cisplatin was used at an optimal dose of 5 *μ*g/ml. The liposomal curcumin was applied to the growth medium at a concentration of 5 *μ*M.

### MTT assay

An MTT assay was applied to determine the viability of HEp-2 cells following various treatments. After 48 h of exposure to the treatment, the cells were incubated with 100 *μ*l MTT (5 mg/ml) solution for 3 h. Subsequently, the MTT solution was replaced with 100 *μ*l dimethyl sulfoxide (DMSO) and 25 *μ*l glycine buffer. The absorbance at 570 nm of untreated HEp-2 cells was considered as 100% cell viability.

### Colony formation assay

Cells of a single-cell suspension (2,000 cells per well) were inoculated in 6-well plates and incubated for 24 h. The cells were cultured with various treatments for 2 weeks. The cells were fixed using ice-cold methanol and stained with crystal violet. Colonies (>50 cells) were analyzed using a Gel-Pro analyzer (Media Cybernetics Inc., Rockville, MD, USA).

### Apoptosis assay

HEp-2 cells were exposed to different treatments for 48 h. The cells were harvested and stained with the Muse Annexin V & Dead Cell assay kit (Merck Millipore, Billerica, MA, USA) according to the manufacturer’s instructions. Cells were analyzed using a Muse™ Cell Analyzer (Merck Millipore). Late apoptosis was defined as Annexin V-positive and propidium iodide (PI)-positive cells.

### Flow cytometry assays and fluorescence-activated cell sorting (FACS) of CD133^+^ cells

Flow cytometry assays for the CD133^+^ cells were performed as previously described (3). In brief, following their respective treatments, the HEp-2 cells were collected and rinsed with PBS. The number of dissociated cells was counted and 1×10^7^ cells were subsequently transferred to 100 *μ*l buffer containing phycoerythrin (PE)-conjugated CD133/2 antibody (Miltenyi Biotec Inc., Auburn, CA, USA) for 30 min and protected from light. The cells were then washed and analyzed using a flow cytometer (BD FACSCalibur; BD Biosciences, Franklin Lakes, NJ, USA). The CD133^+^ and CD133^−^ cells were sorted by FACS as previously described (3).

### Western blotting

Protein extracts from CD133^+^ and CD133^−^ cells were separated using 12% sodium dodecyl sulfate-polyacrylamide gel electrophoresis (SDS-PAGE) and subsequently transferred to a PVDF membrane. Anti-ABCG2 antibody (Santa Cruz Biotechnology, Inc., Dallas, TX, USA) and anti-β-actin antibody (Life Technologies, Grand Island, NY, USA) were used for detection with an ECL Plus system (GE Healthcare, Pittsburgh, PA, USA).

### Statistical analysis

Statistical analysis was performed using SPSS version 16 (IBM, Armonk, NY, USA). Data are presented as the mean ± standard deviation (SD) and evaluated using a t-test. P<0.05 was considered to indicate a statistically significant difference.

## Results

### Curcumin affects the CD133^+^ population in HEp-2 cells

**Figure 1.**
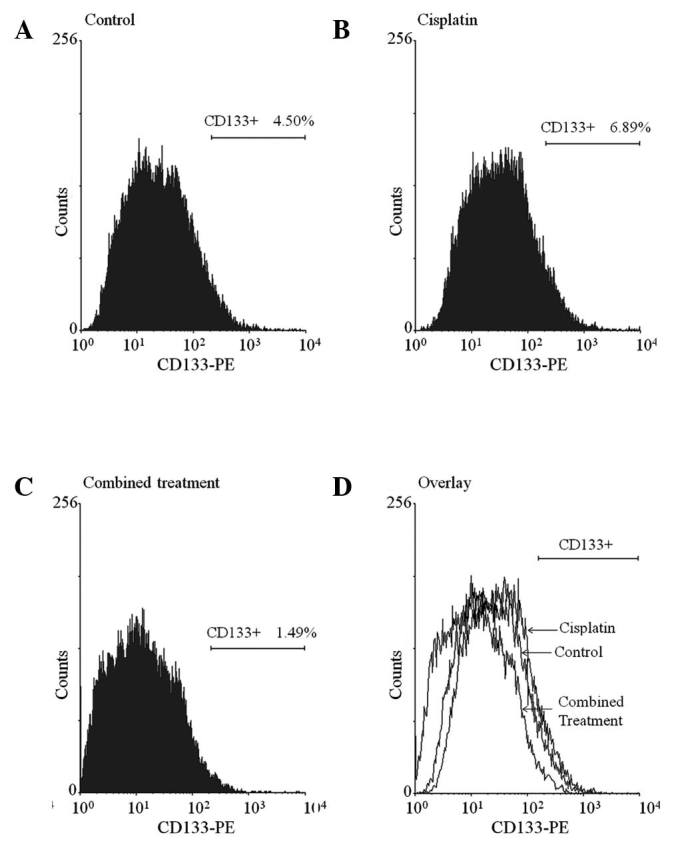
Flow cytometry analysis of the CD133^+^ population. The CD133^+^ population in HEp-2 cells was analyzed by flow cytometry with PE-CD133^+^ antibody. (A) 4.50% CD133^+^ cells were detected among untreated HEp-2 cells. (B) 6.89% CD133^+^ cells were detected in cisplatin-treated cells. (C) 1.45% CD133^+^ cells were detected in HEp-2 cells treatment with a combination of cisplatin and curcumin. (D) An overlay of figures A-C in which the three peaks from left to right indicate combined treatment, untreated control and cisplatin treatment, respectively.

In this study, an average of 4.50% CD133^+^ cells was detected in untreated HEp-2 cells and the CD133^+^ population was increased to 6.89% with cisplatin treatment. However, when cisplatin was applied with curcumin, the CD133^+^ population was markedly reduced to 1.49% (Fig. 1A–C). As shown in Fig. 1D, the over-laid graph displays a clear left shift between the CD133 signals of the cisplatin group and the combined treatment group. This result indicated that cisplatin led to enrichment of the CD133^+^ population in HEp-2 cells and that the enrichment was significantly suppressed by combined treatment with curcumin.

### Curcumin induces apoptosis of HEp-2 cells

**Figure 2.**
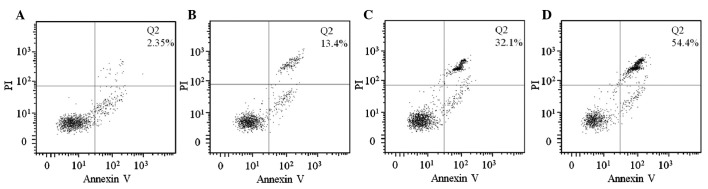
Annexin V/PI apoptosis analysis of HEp-2 cells after treatment. Cell apoptosis was assayed by flow cytometry using the Annexin V/PI method. Late apoptosis was defined as Annexin V-positive and PI-positive cells (Q2). The percentage of apoptotic cells was detected to be (A) 2.35% in untreated HEp-2 cells as a control, (B) 13.4% in curcumin-treated HEp-2 cells, (C) 32.1% in cisplatin-treated HEp-2 cells and (D) 54.4% in HEp-2 cells treated with a combination of curcumin and cisplatin. PI, propidium iodide.

The results of the Annexin V/PI assay demonstrated that curcumin and cisplatin individually induced the apoptosis of HEp-2 cells following 48 h exposure to the drugs and the effect was enhanced when these two drugs were applied simultaneously as a combined treatment. The percentage of late apoptosis in the untreated HEp-2 cells was 2.35% (Fig. 2A, Q2), whereas following treatments with curcumin and cisplatin the percentages of late apoptosis were increased to 13.4 and 32.1%, respectively (Fig. 2B and C, Q2). Following combined treatment with cisplatin and curcumin, the percentage of late apoptosis was significantly increased to 54.4% (Fig. 2D, Q2).

### Curcumin suppresses the proliferation of HEp-2 cells

**Figure 3.**
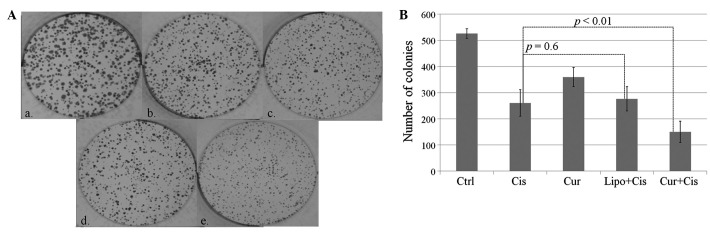
Colony formation assay of HEp-2 cells with different treatments. (A) HEp-2 cells (2000 cells per well) were cultured with different treatments for two weeks and stained with crystal violet. (a) Untreated cells; (b) curcumin-treated cells; (c) cisplatin-treated cells; (d) cells treated with cisplatin and liposomal vehicle; and (e) cells treated with curcumin and cisplatin. (B) Colonies (>100 cells) were analyzed using a Gel-Pro analyzer. Data are presented as the mean of five independent experiments with SD. Statistical significance was indicated by P-values.

The colony formation assay suggested that, combined with cisplatin, curcumin synergistically reduced the clonogenicity and proliferation of HEp-2 cells. Following 2 weeks of incubation, the average number of colonies (>100 cells) in the untreated group was 526 and the number of colonies was reduced in the curcumin and cisplatin groups. When cisplatin was applied with curcumin, the number of colonies was significantly decreased to 150 (Fig. 3A and B). Furthermore, the size of the colonies in the combined treatment group was also reduced (Fig. 3A).

**Figure 4.**
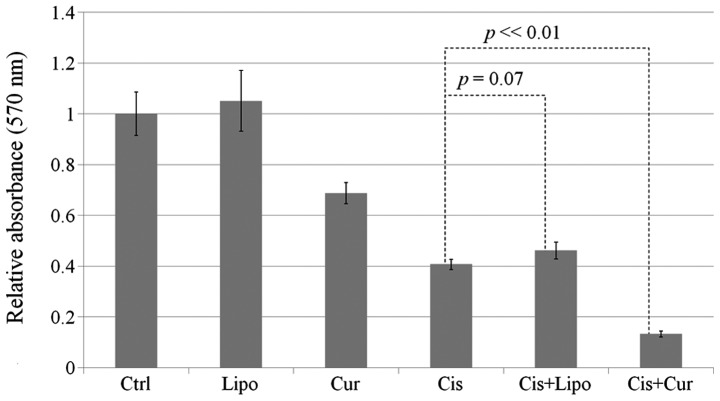
MTT assay of HEp-2 cells following treatment. Cell viabilities after treatments were assessed by MTT assay. The absorbance at 570 nm of untreated HEp-2 cells was considered as 100% (as 1 in the bar chart). Relative absorbances of liposomal vehicle treatment (Lipo), curcumin treatment (Cur), cisplatin treatment (Cis), combined treatment with cisplatin and liposomal vehicle (Cis+Lipo) and combined treatment with cisplatin and curcumin (Cis+Cur) were presented as the mean of five independent experiments with SD. Statistical significance was indicated by P-values. MTT, 3-(4,5-dimethylthiazol-2-yl)-2,5-diphenyltetrazolium bromide.

In addition to the colony formation assay, the MTT assay further confirmed that the combined application of cisplatin and curcumin was able to suppress the viability of HEp-2 cells. The application of cisplatin alone reduced the cell viability ~60%. However, with combined treatment, the reduction was enhanced to ~90% (Fig. 4).

### ABCG2 expression was reduced by curcumin in CD133^+^ cells

**Figure 5.**
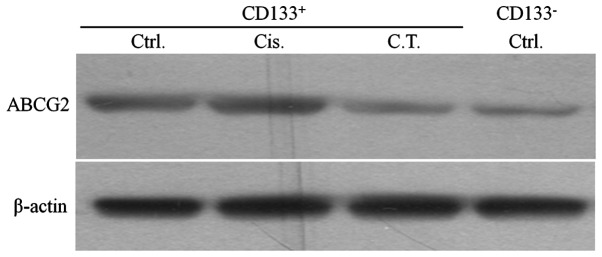
Expression analysis of ABCG2. CD133^+^ and CD133^−^ cells were sorted by FACS sorting and proteins were extracted in each population. The expression levels of ABCG2 were investigated by western blot analysis. In the CD133^+^ population, the expression of ABCG2 was evaluated in untreated CD133^+^ cells (CD133^+^-Ctrl.), cisplatin-treated CD133^+^ cells (CD133^+^-Cis.) and CD133^+^ cells treated with a combination of cisplatin and curcumin (CD133^+^-C.T.). The expression of ABCG2 in untreated CD133^−^ cells (CD133-Ctrl.) was also assayed as a low expression control. β-actin was used as internal control. FACS, fluorescence-activated cell sorting; ABCG2, ATP-binding cassette sub-family G member 2.

The CD133^+^ and CD133^−^ cells were sorted after each treatment and the levels of ABCG2 expression were investigated. No significant difference in the levels of ABCG2 expression was detected between the untreated and cisplatin-treated CD133^+^ cells. However, the expression level was reduced in the combined treatment group and was as low as that in the untreated CD133^−^ group (Fig. 5).

## Discussion

Laryngeal carcinoma is a malignant type of head and neck cancer. Chemotherapy is the main treatment for laryngeal carcinoma and other head and neck cancers; however, major barriers to this therapy, including chemoresistance, have prompted investigation into the underlying mechanisms of anti-cancer chemotherapy and the development of optimal treatment methods (13).

The cancer stem cell theory is one of the most likely explanations for chemoresistance (14). It has been established that CD133 is an important marker for cancer stem cells in the laryngeal carcinoma cell line HEp-2 (4). Furthermore, it has previously been reported that in the HEp-2 cell line, CD133^+^ cancer stem cells are responsible for drug resistance to chemotherapeutic agents (3). In the present study, we attempted to utilize a traditional Chinese medicine and a classical chemotherapy drug as a combined treatment to induce the sensitivity of CD133^+^ stem cells and enhance therapeutic effectiveness.

MTT and colony formation assays demonstrated the anti-cancer effect of curcumin. It reduced the clonogenicity and suppressed the proliferation of HEp-2 cells. The liposomal vehicle had no effect either when used alone or in combination with cisplatin (control, Fig. 4), which indicates that the application of curcumin significantly enhanced the effectiveness of cisplatin. In this study, curcumin induced the apoptosis of HEp-2 cells, consistent with a study reporting that curcumin induced apoptosis in pancreatic carcinoma (12). Moreover, in line with the findings of the MTT assay, curcumin markedly enhanced apoptosis when applied with cisplatin.

In order to investigate the curcumin-enhanced anticancer effect of cisplatin, the chemoresistance of CD133^+^ cancer stem cells, one of the major barriers of cisplatin treatment (15), was studied. In the HEp-2 laryngeal carcinoma cell line, ~1.5–3.5% of cells were previously identified to be CD133^+^ cancer stem cells (3,4). In the present study, 4.50 and 6.89% CD133^+^ cells were detected in untreated and cisplatin-treated HEp-2 cells, respectively. CD133^+^ stem cells were enriched following cisplatin treatment due to the insensitivity of CD133^+^ cells to chemotherapeutic agents. Similar results have also been observed in other cancer treatments (15), and may reflect a problem in the current treatment of laryngeal carcinoma; although chemotherapeutic drugs kill the majority of cancer cells, the cancer stem cells may also lead to drug resistance and tumor recurrence. However, when cisplatin was applied with curcumin, the percentage of CD133^+^ cells was markedly reduced to 1.49%. The enrichment of CD133^+^ stem-like cells was significantly suppressed by combined treatment with curcumin, indicating that curcumin may increase the sensitivity of CD133^+^ cells to cisplatin, leading to the suppression of chemoresistance of HEp-2 cells.

The ATP-binding cassette transporter, ABCG2, functions as an ATP-dependent drug efflux pump contributing to drug resistance (16,17) and is highly expressed in cells with a side population phenotype (18). It has been demonstrated that ABCG2 is one of the most important genes for the chemoresistance of cancer stem cells (19,20). It has been observed that ABCG2 is highly expressed in CD133^+^ HEp-2 cells, leading to chemoresistance (3). In the current study, we demonstrated that combined treatment with cisplatin and curcumin reduced the expression of ABCG2 in CD133^+^ cancer stem cells, indicating that curcumin may suppress ABCG2-induced chemoresistance in CD133^+^ HEp-2 cells.

In conclusion, the present study indicated that the application of curcumin may induce the sensitivity of CD133^+^ cancer stem cells to cisplatin and therefore enhance the effectiveness of cisplatin on the laryngeal carcinoma HEp-2 cell line. The reduced expression of ABCG2 in CD133^+^ cells may be responsible for the induced sensitivity of CD133^+^ cells to cisplatin. The combined application of curcumin with chemotherapeutic drugs may be a reliable and effective approach for the treatment of laryngeal carcinoma. Furthermore, as a major component of the spice turmeric, dietary curcumin may also be used for the prevention of laryngeal carcinoma and other types of cancer. Further studies are required to explore the application of curcumin in the treatment of other cancer types.
